# PTEN pathogenic variants are associated with poor prognosis in patients with advanced soft tissue sarcoma

**DOI:** 10.1038/s44276-023-00029-3

**Published:** 2024-01-30

**Authors:** Minggui Pan, Maggie Y. Zhou, Chen Jiang, Zheyang Zhang, Nam Bui, Jeffrey Bien, Amanda Siy, Ninah Achacoso, Aleyda V. Solorzano, Pam Tse, Elaine Chung, Wenwei Hu, Sachdev Thomas, Kristen Ganjoo, Laurel A. Habel

**Affiliations:** 1 Division of Research, Kaiser Permanente, Oakland, CA 94612 USA; 2 Sarcoma Program, Division of Oncology, Stanford University School of Medicine, Stanford, CA 94305 USA; 3 State Key Laboratory of Cellular Stress Biology, School of Life Sciences, Faculty of Medicine and Life Sciences, Xiamen University, Xiamen, Fujian 361102 China; 4 National Institute for Data Science in Health and Medicine, Xiamen University, Xiamen, Fujian 361102 China; 5 Rutger’s Cancer Institute of New Jersey, New Brunswick, NJ 08903 USA; 6 Department of Oncology and Hematology, Kaiser Permanente, Vallejo, CA 94589 USA

## Abstract

**Background:**

We aimed to examine whether PTEN pathogenic variants (mutPTEN) were associated with overall survival (OS) in patients with advanced soft tissue sarcoma (STS) with the presence of one or more of the most common genomic alterations including *p53, CDKN2A, RB1*, and *ATRX* pathogenic variants.

**Methods:**

This study included patients from Kaiser Permanente Northern California and Stanford Cancer Center with grade 2 or higher locally advanced and metastatic STS.

**Results:**

A total of 174 patients had leiomyosarcoma (LMS), 136 had undifferentiated pleomorphic sarcoma (UPS), 78 had Liposarcoma (LPS), and 214 had other histology subtypes (Others). Among all patients with STS, OS was worse for those with mutPTEN versus wild-type PTEN (wtPTEN, adjusted HR [aHR] = 1.58 [95% CI, 1.11–2.23]), mutCDKN2A vs wtCDKN2A (aHR = 1.33 [95% CI .99–1.80]), and mutRB1 vs wtRB1 (aHR = 1.26 [95% CI 0.93–1.70[), while OS was similar for mutp53 vs wtp53 and mutATRX vs wtATRX. MutPTEN versus wtPTEN was consistently associated with worse OS in histologic subtypes including LMS and UPS and molecular subgroups.

**Conclusion:**

MutPTEN vs wtPTEN was associated with worse OS in advanced STS. If confirmed, our findings could be helpful for prognostic stratification in clinical practice and for further understanding the molecular mechanisms of STS.

## Introduction

Soft tissue sarcomas (STSs) are a group of relatively uncommon malignancies that are characterized by heterogeneity, with leiomyosarcoma (LMS), undifferentiated pleomorphic sarcoma (UPS), and Liposarcoma (LPS) the most common histologic subtypes [1]. Several dozen genomic alterations have been identified in advanced STSs including fusions, deletions, missense pathogenic variants, and others [2, 3]. Different histologic subtypes share some common genomic alterations but also harbor unique alterations. For example, *MDM2* and *CDK4* amplifications are universally present in dedifferentiated liposarcoma, while *p53* (mutp53)*, CDKN2A* (mutCDKN2A), *RB1* (mutRB1), *PTEN* (mutPTEN) and *ATRX* (mutATRX) deletions or pathogenic variants are the most common alterations in LMS and UPS [2–4]. Other histologic subtypes, such as synovial sarcoma, are characterized by a unique fusion gene SS18-SSX [5, 6].

The treatment options for advanced STSs have evolved only modestly over the past three decades with very limited options still available [7–10]. Success with systemic therapy has been far from satisfactory for the most part [1, 11–13]. The recent advances with immunotherapy including immune checkpoint inhibitors and adoptive cell therapy have shown only modest efficacy [13–19]. The prognosis for patients with intermediate to high-grade STSs remains very poor. How the common genomic alterations affect the prognosis of advanced STSs has not been well studied. Previous studies have suggested that mutp53 is associated with poorer disease-free survival (DFS) and overall survival (OS) [20, 21]. Other studies have suggested that mutCDKN2A is associated with poorer OS [8, 22]. Little is known about the potential impact of mutPTEN, mutRB1 and mutATRX on the prognosis of STS. The current prognostic stratifications for STS remain dependent on clinical factors [1, 23, 24].

The role of mutPTEN in the prognosis of advanced STS remains unclear. In this study, we examined genomic pathogenic variants and OS among a combined cohort of 602 adult patients with advanced STS from Kaiser Permanente Northern California (KPNC) and Stanford Cancer Center using next-generation sequencing (NGS) data, with focus on the role of mutPTEN in the presence of co-pathogenic variants including mutp53, mutCDKN2A, mutRB1, and mutATRX.

## Materials and methods

### Study population

Our dataset included a total of 602 eligible patients (297 from KPNC and 305 from Stanford Cancer Center) with grade 2 or higher locally advanced (unresectable) or metastatic STS [25]. The KPNC cohort had NGS performed using StrataNGS (Ann Arbor, Michigan) from November 2017 to June 2022 and the Stanford cohort had NGS performed from February 2015 to April 2022 (see below). Patient data on demographics, Charlson comorbidity index (CCI), performance status (PS), and receipt of systemic therapy were obtained from the electronic medical record (Epic) and cancer registry database. CCI was based on the 12-month period prior to diagnosis of locally advanced or metastatic STS. This study was approved by the KPNC and Stanford Cancer Center institutional review boards with a waiver of consent.

### NGS

StrataNGS is currently a 429-gene, pan-solid tumor, NGS assay for formalin-fixed paraffin-embedded (FFPE) tumor tissue, performed on co-isolated DNA and RNA [26]. *ATRX* was included in StrataNGS panel in August 2020. For the Stanford cohort, NGS was performed by FoundationOne (Foundation Medicine, Cambridge, MA) [27, 28], Tempus (Tempus Labs, Inc., Chicago, IL) [28, 29], and Altera™ (Natera, Inc., Austin, TX) [30]. All platforms evaluated the most common genomic alterations of interest, including *p53, CDKN2A, RB1, PTEN and ATRX*. MutCDKN2A includes CDKN2A deletion, pathogenic variants, *CDK4* and *CCND1* amplification. Approximately 60 genes with pathogenic variants were identified. The NGS assays were performed using three multiplexed PCR-based panels (two DNA and one RNA), simultaneously assessed single nucleotide variant, short indels, short structural variants and copy number variation, and all variant classes were analyzed and subjected to independent quality control metrics and bioinformatics pipelines for reporting following the American College of Medical Genetics and Genomics (ACMG) criteria.

### Histology

We included only grade 2 or higher locally advanced (unresectable) or metastatic STS (either de novo or recurrent). We excluded gastrointestinal stromal tumor, benign histology, and grade 1 histology such as well-differentiated LPS, low-grade myxoid LPS, etc. Bone sarcoma was excluded from this analysis. We classified all patients into four histology subtypes to facilitate analysis: LMS, which included uterine LMS (uLMS) and extra-uterine LMS (extra-uLMS), UPS, LPS, and other histology subtypes (Others). LPS included pleomorphic LPS, grade 2 and 3 myxoid LPS and dedifferentiated LPS. UPS included unspecified high-grade sarcoma. The histologic subtypes of Others are shown in Data Supplement (Table A1).

### Treatment

Treatment included the administration of chemotherapy, targeted therapeutics (such as pazopanib) and checkpoint inhibitors after a patient was diagnosed with locally advanced or metastatic disease, either alone or in a certain combination.

### Definition of hotspot TP53 pathogenic variants

We previously defined mutp53 R175H, R248Q, R248W, R249S, R273H, R273L, and R282W as gain-of-function pathogenic variants based on our literature review [31]. The literature on these pathogenic variants were predominantly in carcinoma cell lines and animal models. There is a lack of studies of these pathogenic variants in sarcoma cell lines and animal models; therefore, the functions of these pathogenic variants in sarcoma remain unclear. For this reason, in our sarcoma studies, we have included all missense pathogenic variants involving six hotspot pathogenic variant codons (R175, G245, R248, R249, R173, and W282) as “hotspot pathogenic variants” instead of gain-of-function pathogenic variants. The rest of TP53 pathogenic variants were grouped as non-hotspot pathogenic variants. There were 33 patients with hotspot pathogenic variant.

### Statistical analysis

OS was measured from the date of diagnosis of locally advanced or metastatic STS to the date of death or end of study follow-up (July 28, 2022 for KPNC cohort and December 27, 2022 for Stanford cohort), whichever came first. We used Pearson’s *χ*^2^ test to assess differences in distributions of demographic and clinical factors and in *p53*, *CDKN2A*, *RB1, PTEN* and *ATRX* pathogenic variants. We used the one-way ANOVA test to assess differences in continuous variables. We used the Kaplan–Meier plot (log-rank test) to perform unadjusted (univariate) OS analysis and to estimate the median OS. The number of patients at risk in the Kaplan–Meier OS curves accounted for delayed entry into the cohort at the time of receipt of NGS results (i.e., left-truncation, with median study entry of 7.7 months post-diagnosis) [32]. Cox proportional hazards regression models were used to estimate the adjusted hazard ratio (aHR) and 95% confidence intervals (CI) for the association between pathogenic variant subsets and OS, adjusted for covariates. Time since diagnosis of advanced STS was the time scale used in the regression models, allowing for delayed entry into the cohort [32]. Covariates included in our main regression models (and unless otherwise stated) were age (continuous), sex (male, female), ethnicity (Non-Hispanic White, Black, Asian, Hispanic, other/unknown), PS (0–1, 2–4), CCI (continuous), and treatment received (yes, no). We examined the effect of specific pathogenic variants in a model that included all five pathogenic variants simultaneously: *p53* [yes, no], *CDKN2A* [yes, no], *RB1* [yes, no], *PTEN* (yes, no), and *ATRX* (yes, no, unknown) pathogenic variants, as well as four different histologic subtypes including LMS (yes, no), UPS (yes, no), LPS (yes, no), and Others (yes, no). We conducted subgroup analyses for the effect of each specific pathogenic variant with or without other specified co-pathogenic variants. The statistical analysis was performed using SAS software version 9.4, R (R Core Team, 2020).

## Result

### Demographic and clinical characteristics of the cohort by histology and pathogenic variant status

Mutp53 was detected in 76.4% of patients with LMS, 59.6% with UPS, 15.4% with LPS and 32.7% with Others. The percent of patients with mutCDKN2A, mutRB1, mutPTEN or mutATRX also varied by histologic subtype (Table 1). Patients with mutPTEN had higher percentages of mutRB1, mutp53, and mutCDKN2A compared to patients with wtPTEN (Table 2). Patients with mutp53 versus wtp53 were generally older and more commonly female. In addition, the histology of their tumors was more commonly LMS, had fewer mutCDKN2A but more mutPTEN, mutRB1, and mutATRX (data supplement, Table A2). The demographic characteristics of patients with hotspot vs. non-hotspot mutp53 were not substantially different (Table A3). Patients with mutCDKN2A versus wild-type *CDKN2A* (wtCDKN2A) were generally older, with lower percentage of females, had a higher CCI, had more UPS or LMS, and fewer mutp53, mutRB1, mutPTEN or mutATRX (Data supplement, Table A4). Patients with mutRB1 versus wild-type *RB1* (wtRB1) had a higher percentage of female patients and had a better CCI, and was essentially mutually exclusive with mutCDKN2. In contrast, tumors of patients with mutRB1 had a higher percentage of mutPTEN and mutATRX (Data supplement, Table A5). The histology of tumors of patients with mutPTEN versus wild-type *PTEN* (wtPTEN) was more likely to be LMS and had mutRB1 and mutp53 (Data supplement, Table A5). The histology of tumors of patients with mutATRX versus wild-type *ATRX* (wtATRX) were more commonly LMS, and had higher percentage of mutp53 and mutRB1 but lower percentage of mutCDKN2A (Data supplement, Table A6).

**Table 1:** Demographics by histologic subtypes.

		LMS (*n* = 174)	UPS (*n* = 136)	LPS (*n* = 78)	Others (*n* = 214)	*P* value
Median age		59 (23–88)	63 (22–97)	63 (35–90)	57 (19–94)	<0.001
Female		133 (76.4)	72 (52.9)	37 (47.4)	108 (50.5)	<0.001
Race	Asian	35 (20.1)	22 (16.2)	14 (17.9)	44 (20.6)	0.18
	Black	11 (6.3)	5 (3.7)	2 (2.6)	7 (3.3)	
	Hispanic	26 (14.9)	12 (8.8)	16 (20.5)	39 (18.2)	
	White	99 (56.9)	89 (65.4)	44 (56.4)	115 (53.7)	
	Others	3 (1.7)	8 (5.9)	2 (2.6)	9 (4.2)	
PS	0–1	155 (89.1)	110 (80.9)	63 (80.8)	194 (90.7)	0.01
	2–4	10 (5.7)	21 (15.4)	12 (15.4)	13 (6.1)	
	Unknown	19(5.2)	15(3.7)	3 (3.8)	7 (3.3)	
CCI		1 (0–9)	2 (0–9)	1 (0–7)	1 (0–7)	<0.001
Treatment	Yes	146 (83.9)	103 (75.7)	52 (66.7)	173 (80.8)	0.01
	No	28 (16.1)	33 (24.3)	26 (33.3)	41 (19.2)	
*TP53*	wt	41 (23.6)	55 (40.5)	66 (76.9)	144 (67.3)	<0.001
	mut	133 (76.4)	81 (59.5)	12 (23.1)	70 (32.7)	
*CDKN2A*	wt	166 (95.4)	100 (73.5)	20 (25.6)	177 (82.7)	<0.001
	mut	8 (4.6)	36 (26.5)	58 (74.4)	37 (17.3)	
*RB1*	wt	96 (55.2)	109 (80.1)	69 (88.5)	197 (92.1)	<0.001
	mut	78 (44.8)	27 (19.9)	9 (11.5)	17 (7.9)	
*PTEN*	wt	142 (81.6)	124 (91.2)	74 (94.9)	205 (95.8)	<0.001
	mut	32 (18.4)	12 (8.8)	4 (5.1)	9 (4.2)	
*ATRX*	wt	85 (48.9)	96 (70.6)	52 (66.7)	147 (68.7)	<0.001
	mut	34 (19.5)	14 (10.3)	5 (6.4)	14 (6.5)	
	unknown	55 (31.6)	26 (19.1)	21 (26.9)	53 (24.8)	

The number inside the parenthesis represents the percent except for median age.

*PS* performance status, *CCI* Charlson comorbidity index, *LMS* leiomyosarcoma, *UPS* undifferentiated pleomorphic sarcoma, *LPS* liposarcoma.

**Table 2:** Demographic and clinical characteristics of patients with PTEN pathogenic variant (mutPTEN) vs wild-type PTEN (wtPTEN).

		Wt (*n* = 545)	Mut (*n* = 57)	*P*
Median age		60 (19–92)	56 (35–81)	0.56
Female		315 (57.8)	35 (61.4)	0.61
Race	Asian	106 (19.4)	9 (15.8)	0.06
	Black	19 (3.5)	6 (10.5)	
	Hispanic	84 (15.4)	9 (15.8)	
	White	314 (57.5)	33 (57.9)	
	Others	22 (4.0)	0	
PS	0–1	474 (87.0)	48 (84.2)	0.71
	2–4	49 (9.0)	7 (12.3)	
	Unknown	22 (4.0)	2 (3.5)	
CCI		1 (0–9)	2 (0–6)	0.07
Treatment	Yes	429 (78.7)	45 (78.9)	0.97
	No	116 (21.3)	12 (21.2)	
Histology	LMS	142 (26.1)	32 (56.1)	<0.001
	UPS	124 (22.8)	12 (21.1)	
	LPS	74 (13.6)	4 (7.0)	
	Others	205 (37.6)	9 (15.8)	
*RB1*	wt	440 (80.7)	31 (54.4)	<0.001
	mut	105 (19.3)	26 (45.6)	
*TP53*	wt	294 (53.9)	12 (21.1)	<0.001
	mut	251 (46.1)	45 (78.9)	
*CDKN2A*	wt	410 (75.2)	53 (93.0)	0.002
	mut	135 (24.8)	4 (7.0)	
*ATRX*	wt	349 (64.0)	31 (54.4)	0.30
	mut	58 (10.6)	9 (15.8)	
	unknown	138 (25.3)	17 (29.8)	

The number inside the parenthesis represents the percent except for median age.

*PS* performance status, *CCI* Charlson comorbidity index, *LMS* leiomyosarcoma, *UPS* undifferentiated pleomorphic sarcoma, *LPS* liposarcoma.

### MutPTEN vs wtPTEN was associated with a worse prognosis in the full cohort

**Fig. 1:**
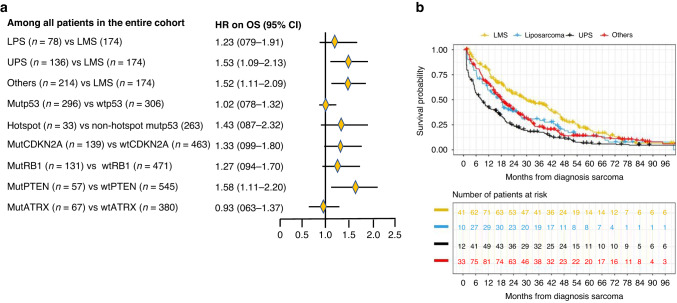
**a** Forest plot of adjusted hazard ratios of OS associated with histologic subtypes and for wild-type vs pathogenic variant comparisons in single genes. aHR adjusted hazard ratio, OS overall survival, LMS leiomyosarcoma, UPS undifferentiated pleomorphic sarcoma, LPS liposarcoma, Others other histology subtypes than LMS, UPS and LPS, Mut pathogenic variant, Wt wild-type. **b** Kaplan–Meyer OS curves of four different histology subtypes. Histology subtypes include uLMS and extra-uLMS; UPS undifferentiated pleomorphic sarcoma, Liposarcoma (including dedifferentiated liposarcoma, pleomorphic liposarcoma, and grade 2 and 3 myxoid liposarcoma), Others (including all other grade 2 and 3 histology subtypes than LMS, UPS, or liposarcoma). The number of patients at risk accounted for left-truncation. Patients who were still alive by the data lock date were censored.

When examining single genes, OS appeared to be worse for patients with mutPTEN versus wtPTEN (aHR = 1.58 [95% CI, 1.11–2.23]), and for patients with mutCDKN2A versus wtCDKN2A (aHR = 1.33 [95% CI 0.99–1.80]), mutRB1 versus wtRB1 (aHR = 1.26 [95% CI 0.93–1.70]). In contrast, mutATRX versus wtATRX did not appear to be associated with worse OS (aHR = 0.93 [95% CI 0.63–1.37]) (Fig. 1a). While mutp53 versus wtp53 was also not associated with OS (aHR = 1.02 [95% CI 0.78–1.32]), hotspot versus non-hotspot mutp53 did appear to be associated with worse OS (aHR = 1.43 [95% CI 0.87–2.32]) (Fig. 1a).

Compared to patients with LMS, OS appeared to be worse for patients with LPS (aHR = 1.22 [95% CI 0.78–1.91]), UPS (aHR = 1.53 [95% CI 1.11–2.14]) or with Other subtypes (aHR = 1.52 [95% CI 1.11–2.14]). (Fig. 1a). Median OS was 9.2 months for UPS, 15.4 months for LS, 14.4 for Other subtypes and 29.5 months for LMS, respectively (Fig. 1b).

### MutPTEN vs wtPTEN was associated with worse OS in LMS and UPS

#### LMS

**Fig. 2:**
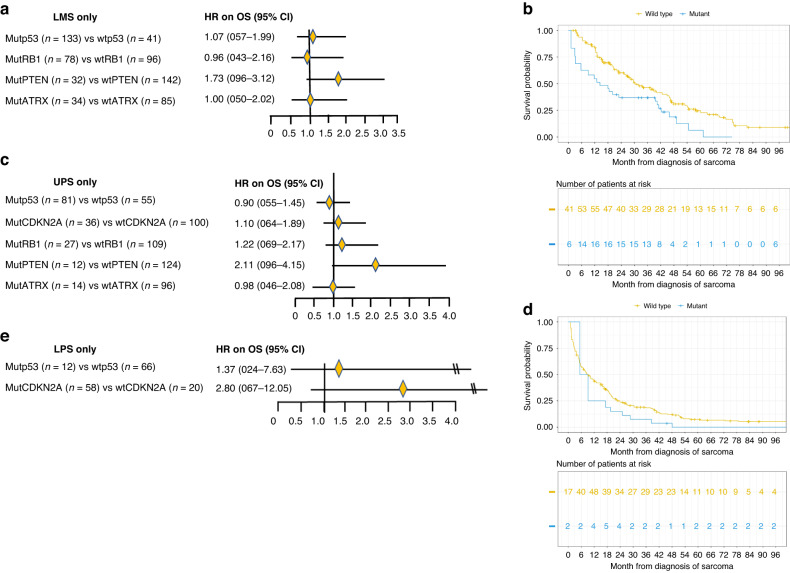
**a** Forest plot of hazard ratios of OS for patients with leiomyosarcoma (LMS). aHR adjusted hazard ratio, OS overall survival, uLMS uterine leiomyosarcoma, extra-uLMS extra-uterine leiomyosarcoma, Mut pathogenic variant, Wt wild-type. **b** Kaplan–Meir OS curves of LMS patients with mutPTEN versus with wtPTEN. The number of patients at risk accounted for left-truncation. Patients who were still alive by the data lock date were censored. **c** Forest plot of hazard ratios of OS for patients with undifferentiated pleomorphic sarcoma (UPS). aHR adjusted hazard ratio, OS overall survival, Mut pathogenic variant, Wt wild-type. **d** Kaplan–Meir OS curves of UPS patients with mutPTEN vs with wtPTEN. The number of patients at risk accounted for left-truncation. Patients who were still alive by the data lock date were censored. **e** Forest plot of adjusted hazard ratios of OS for patients with liposarcoma (LPS). aHR adjusted hazard ratio, OS overall survival, Mut pathogenic variant, Wt wild-type.

Among patients with LMS, mutPTEN versus wtPTEN was associated with substantially worse OS (aHR = 1.73 [95% CI 0.96–3.12]) (Fig. 2a), with median OS of 14 versus 32 months (Fig. 2b), while mutp53 versus wtp53 (aHR = 1.07 [95% CI 0.57–1.99]), mutRB1 versus wtRB1 (aHR = 0.96 [95% CI 0.43–2.16]), mutATRX versus wtATRX (aHR = 1.00 [95% CI 0.50–2.02]) were not associated with substantial OS difference.

#### UPS

Among patients with UPS, OS with mutPTEN versus wtPTEN (HR = 2.11 [95% CI 0.96–4.15]) was substantially worse (Fig. 2c), with a median OS of 5.5 versus 9.0 months (Fig. 2d). However, the sample was small. OS with mutp53 versus wtp53 (aHR = 0.90 [95% CI 0.55–1.45]), OS with mutCDKN2A versus wtCDKN2A (aHR = 1.10 [95% CI 0.64–1.89]), mutRB1 versus wtRB1 (aHR = 1.22 [95% CI 0.69–2.17]), mutATRX versus wtATRX (HR = 0.98 [95% CI 0.46–2.08]) were not substantially different.

#### LPS

Among patients with LPS, OS with mutp53 versus wtp53 appears slightly worse (aHR = 1.37 [0.24–7.63]) but the sample size with mutp53 was small (*n* = 12) (Fig. 2e). OS with mutCDKN2A versus wtCDKN2A was worse (aHR = 2.80 [95% CI 0.67–12.05]) (Fig. 2e). The sample size with mutRB1, mutPTEN and mutATRX was too small to allow meaningful analysis.

#### Others

Among patients with Others, OS with mutPTEN versus wtPTEN (aHR = 1.50 [95% CI 0.68–3.31]) was worse, and OS with mutp53 versus wtp53 was also worse (aHR = 1.47 [95% CI 0.91–2.39]). OS with mutCDKN2A versus wtCDKN2A (aHR = 0.99 [95% CI 0.61–1.59]) and mutRB1 versus wtRB1 (aHR = 0.95 [95% CI 0.42–1.11]) were not substantially different, and OS with mutATRX versus wtATRX (aHR = 0.59 [95% CI 0.23–1.49]) was better but had small sample size (Data supplement, Fig. A1).

### Association of mutp53 vs wtp53 with OS among subgroups based on co-pathogenic variants

**Fig. 3:**
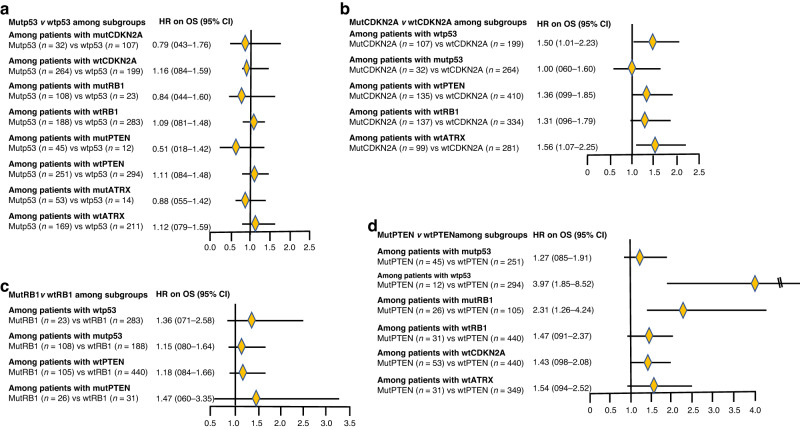
**a** Forest plot of adjusted hazard ratios of OS for patients with mutp53 versus wtp53 among subgroups. aHR adjusted hazard ratio, OS overall survival, Mut pathogenic variant, Wt wild-type. **b** Forest plot of adjusted hazard ratios of OS for patients with mutCDKN2A versus wtCDKN2A among subgroups. aHR adjusted hazard ratio, OS overall survival, Mut pathogenic variant, Wt wild-type. **c** Forest plot of adjusted hazard ratios of OS for patients with mutRB1 versus wtRB1 among subgroups. aHR adjusted hazard ratio, OS overall survival, Mut pathogenic variant, Wt wild-type. **d** Forest plot of adjusted hazard ratios of OS for patients with mutPTEN versus wtPTEN among subgroups. aHR adjusted hazard ratio, OS overall survival, Mut pathogenic variant, Wt wild-type.

Although many subgroups were small, there was a suggestion that mutp53 versus wtp53 was associated with modestly better OS among patients with mutCDKN2A (aHR = 0.79 [95% CI 0.43–1.76]) but not with wtCDKN2A (aHR = 1.16 [95% CI 0.84–1.59]), and among patients with mutRB1 (aHR = 0.84 [95% CI 0.44–1.60]) but not among patients with wtRB1 (HR = 1.09 [95% CI 0.81–1.48]), and among patients with mutPTEN (aHR = 0.51 [95% CI 0.18–1.42]) but not among patients with wtPTEN (aHR = 1.11 [95% CI 0.84–1.48]), and among patients with mutATRX (aHR = 0.88 p95% CI 0.55–1.42]) but not among patients with wtATRX (aHR = 1.12 [95% CI 0.79–1.59]) (Fig. 3a).

### Association of mutCDKN2A vs. wtCDKN2A with OS among subgroups based on co-pathogenic variants

MutCDKN2A versus wtCDKN2A was associated with substantially worse OS in among patients with wtp53 (aHR = 1.50 [95% CI 1.01–2.23]), among patients with wtPTEN (aHR = 1.36 [95% CI 0.99–1.85]), among patients with wtRB1 (aHR = 1.31 [95% CI 0.96–1.79), and among patients with wtATRX (aHR = 1.56 [95% CI 1.07–2.25]) (Fig. 3b). but not among patients with mutp53 (aHR = 1.00 [95% CI 0.60–1.60]) (Fig. 3b).

### Association of mutRB1 vs wtRB1 with OS among subgroups based on co-pathogenic variants

MutRB1 versus wtRB1 was associated with modestly worse OS among patients with wtp53 (aHR = 1.36 [95% CI 0.71–2.58]) and among patients with mutPTEN (aHR = 1.47 [95% CI 0.60–3.35]), but slightly worse OS among patients with mutp53 (aHR = 1.15 [95% CI 0.80–1.64]), and among patients with wtPTEN (aHR = 1.18 [95% CI 0.84–1.66]) (Fig. 3c).

### Association of mutPTEN vs wtPTEN with OS among subgroups based on co-pathogenic variants

MutPTEN versus wtPTEN was associated with slightly worse OS among patients with mutp53 (aHR = 1.27 [95% CI 0.85–1.91]) but substantially worse OS among patients with wtp53 (aHR = 3.97 [95% CI 1.85–8.52]) (Fig. 3d). OS with mutPTEN versus wtPTEN was substantially worse among patients with mutRB1 (aHR = 2.31 [95% CI 1.26–4.24]), and worse among patients with wtRB1 (aHR = 1.47 [95% CI 0.91–2.37]) and among patients with wtCDKN2A (aHR = 1.43 [95% CI 0.98–2.08]) and among patients with wtATRX (aHR = 1.54 [95% CI 0.94–2.52]) (Fig. 3d).

## Discussion

In this study using the combined KPNC and Stanford cohort of 602 patients with advanced STS, we have shown that mutPTEN vs wtPTEN was associated with worse OS. We have also shown that histologically LMS had the best while UPS had the worst OS. It appeared that hotspot versus non-hotspot mutp53 but not mutp53 versus wtp53 was associated with worse OS; In addition, there was a suggestion that mutp53 versus wtp53 was associated with favorable OS among subgroups with mutCDKN2A, mutRB1, mutPTEN and mutATRX than among subgroups with wtCDKN2A, wtRB1, wtPTEN and wtATRX; Also, mutCDKN2A versus wtCDKN2A, mutRB1 versus wtRB1 and mutPTEN versus wtPTEN were associated with worse OS primarily among patients with wtp53 but not among patients with mutp53.

MutPTEN is a common pathogenic variant in advanced STS. To our knowledge, our study is the first to reveal that mutPTEN versus wtPTEN was associated with worse OS. MutPTEN versus wtPTEN was consistently associated with worse OS among the entire cohort, among histologic subtypes including LMS and UPS and among several molecular subgroups including patients with wtp53, wtCDKN2A, wtATRX, and mutRB1. This finding should be helpful for clinical practice in stratifying prognosis and potentially be helpful in subgroup stratification in clinical trials. Our findings are consistent with some of the previous reports on PTEN pathogenic variant and prognosis in other malignancies [33–35].

The worse OS associated with hotspot versus non-hotspot mutp53 appears to be consistent with our previous studies in metastatic colorectal cancer (CRC) and in advanced pancreatic ductal adenocarcinoma (PDAC) [31, 36]. However, the attenuation of the adverse OS effect of mutCDKN2A, mutRB1 and mutPTEN by mutp53 was unexpected and intriguing, though independently for others, mutp53 was associated with substantially worse OS. These results suggest that different advanced STS may be driven by driver pathogenic variant and co-pathogenic variants differently during the evolution of disease despite similar pathogenic variant profiles being present; some may adopt p53 pathogenic variant as a driver pathogenic variant and PTEN pathogenic variant as a co-pathogenic variant of passenger in nature, while some may adopt PTEN (or RB1, or CDKN2A or others) pathogenic variant as a driver pathogenic variant while p53 pathogenic variant as a subsequent pathogenic variant acting more of a passenger pathogenic variant, resulting in diverse biology with prognostic biases. Studies in STS on the association of mutp53 with prognosis have been limited. In the pooled analysis of MOSCATO and ProfiLER precision trials in sarcoma, mutp53 was found to be associated with worse DFS, but better response to anthracycline chemotherapy [20]. A previous study showed worse OS with mutp53 versus wtp53 in patients with metastatic sarcoma based on univariate analysis with a small sample size [21]. A small retrospective study with only 19 patients showed that mutp53 was predictive of response to pazopanib in patients with advanced STS.33 We speculate that certain *TP53* pathogenic variants may present a vulnerability in STS for treatment targeting, similar to some of the well-characterized genomic lesions that have been targeted [37–41]. This notion appears consistent with the above-referenced studies showing that mutp53 was associated with a better response to doxorubicin and pazopanib [20, 33]. Certain chemical agents such as arsenic trioxide have been shown to be capable of restoring the conformation of multiple different p53 mutants into their original tumor suppressive function, indicating that mutant p53 proteins could be molecularly “differentiated” or “rescued” [42–45]. In our previous study in patients with metastatic CRC, mutp53 overall was not associated with prognosis, similar to our current finding in advanced STS; however, hotspot mutp53 was associated with worse OS than non-hotspot mutp53 in left-sided CRC (LCC) but not in right-sided CRC (RCC), while RCC was associated with worse OS than LCC only in patients with non-hotspot TP53. Our current results with advanced STS appear consistent with our previous findings in metastatic CRC as RCC and LCC possess distinct histologic characteristics [31]. The mechanisms of such a co-pathogenic variant-dependent OS differential would be interesting to investigate. This may be explored by functional studies and in prospective trials.

Our results showing that OS of mutCDKN2A versus wtCDKN2A and OS of mutPTEN versus wtPTEN were worse among patients whose STS retained wtp53 but not among patients whose tumor harbored mutp53 are also consistent with the results that mutp53 versus wtp53 was associated with better OS among patients with mutCDKN2A, mutRB1 and mutPTEN. This suggests that mutp53 could be functionally capable of overcoming the adverse effect by mutCDKN2A or mutPTEN in certain aspects. This would be quite intriguing if confirmed in additional studies as mutCDKN2A and mutPTEN would usually be considered cooperative with mutp53 to exacerbate cancer progression [30, 46–49], though better outcomes associated with mutp53 over wtp53 have been presented in some previous studies. In a study on patients with non-small cell lung cancer (NSCLC) bearing an *STK11* pathogenic variant (mutSTK11), it was found that mutp53 conferred better OS over wtp53 [50]. In a separate study on patients with NSCLC bearing a mutSTK11, it was found that mutp53 was associated with better PFS and immunologically metabolic reprogramming compared to wtp53 [51]. Experimentally, a recent study showed that wtp53 could promote mitotic bypass and whole genome duplication under cyclin E-induced replicative stress, suggesting that wtp53 could exacerbate cancer progression under certain biological circumstances [52]. This finding brought about a novel concept on the roles of *TP53* that warrants further investigations in animal and human studies.

Our study has several strengths. To our knowledge, our sample size is the largest compared to all the published STS genomic studies cohorts. In addition, our cohort includes patients from two large institutions with multi-specialty care centers. Furthermore, our cohort included the five most commonly mutated genes and the four most common histology subtypes in STS. We used Cox regression modeling to adjust for several patient and clinical variables. Our study also has limitations. First, it is a retrospective study and some patients did not have NGS performed at the time of diagnosis of advanced disease until months later. Nonetheless, we used appropriate statistical methods to address this issue [32]. Second, only approximately two-third of patients had known ATRX pathogenic variant status. Third, the sample size of some subgroups was small and analyses should be considered exploratory. Fourth, the results with Others could be more challenging to interpret as it is comprised of heterogeneous STS subtypes.

In summary, our study suggests that mutPTEN was associated with worse OS compared to wtPTEN and mutp53 appeared to attenuate the adverse OS effect by mutCDKN2A and mutPTEN co-pathogenic variants in advanced STS. If confirmed, our results provide new insight for further understanding the molecular mechanisms of STS and may improve prognostic stratifications in clinical practice and future clinical trial designs based on molecular subgroups and histologic subtypes.

## Supplementary information

Data supplement

## Data Availability

Kaiser Permanente Northern California (KPNC) Institutional Review Board has not provided approval for StrataNGS data on individual patients used in this study to be placed in a public access repository. However, researchers can request access to use this study data by contacting the DOR Data Sharing Workgroup at DOR-DataSharingWorkgroup@kp.org.

## References

[1] Gamboa AC, Gronchi A, Cardona K. Soft-tissue sarcoma in adults: an update on the current state of histiotype-specific management in an era of personalized medicine. CA Cancer J Clin. 2020;70:200–29. 10.3322/caac.21605 32275330 10.3322/caac.21605

[2] Cancer Genome Atlas Research Network. Comprehensive and integrated genomic characterization of adult soft tissue sarcomas. Cell. 2017;171:950–65.e28. 10.1016/j.cell.2017.10.014 29100075 10.1016/j.cell.2017.10.014 PMC5693358

[3] Nacev BA, Sanchez-Vega F, Smith SA, Antonesc CR, Rosenbarm E, Shi H, et al. Clinical sequencing of soft tissue and bone sarcomas delineates diverse genomic landscapes and potential therapeutic targets. Nat Commun. 2022;13:3405. 10.1038/s41467-022-30453-x 35705560 10.1038/s41467-022-30453-x PMC9200818

[4] Barretina J, Taylor BS, Banerji S, Ramo AH, Lagos-Quintana M, Decarolis PL, et al. Subtype-specific genomic alterations define new targets for soft-tissue sarcoma therapy. Nat Genet. 2010;42:715–21. 10.1038/ng.619 20601955 10.1038/ng.619 PMC2911503

[5] Kadoch C, Crabtree GR. Reversible disruption of mSWI/SNF (BAF) complexes by the SS18-SSX oncogenic fusion in synovial sarcoma. Cell. 2013;153:71–85. 10.1016/j.cell.2013.02.036 23540691 10.1016/j.cell.2013.02.036 PMC3655887

[6] Kadoch C, Hargreaves DC, Hodges C, Elia L, Ho L, Ranish J, et al. Proteomic and bioinformatic analysis of mammalian SWI/SNF complexes identifies extensive roles in human malignancy. Nat Genet. 2013;45:592–601. 10.1038/ng.2628 23644491 10.1038/ng.2628 PMC3667980

[7] Seddon B, Strauss SJ, Whelan J, Leahy M, Woll P, Cowie F, et al. Gemcitabine and docetaxel versus doxorubicin as first-line treatment in previously untreated advanced unresectable or metastatic soft-tissue sarcomas (GeDDiS): a randomised controlled phase 3 trial. Lancet Oncol. 2017;18:1397–410. 10.1016/S1470-2045(17)30622-8 28882536 10.1016/S1470-2045(17)30622-8 PMC5622179

[8] Pan M, Trieu MK, Sidhu M, Yu J, Seto T, Ganjoo K. Fourteen-day gemcitabine-docetaxel chemotherapy is effective and safer compared to 21-day regimen in patients with advanced soft tissue and bone sarcoma. Cancers (Basel). 2021;13:1983. 10.3390/cancers13081983 10.3390/cancers13081983 PMC8074251 33924080

[9] Demetri GD, von Mehren M, Jones RL, Hensley M, Schuietze SM, Staddon A, et al. Efficacy and safety of trabectedin or dacarbazine for metastatic liposarcoma or leiomyosarcoma after failure of conventional chemotherapy: results of a phase III randomized multicenter clinical trial. J Clin Oncol. 2016;34:786–93. 10.1200/JCO.2015.62.4734 26371143 10.1200/JCO.2015.62.4734 PMC5070559

[10] van der Graaf WT, Blay JY, Chawla SP, Kim B, Bui-Nguyen B, Casali P, et al. Pazopanib for metastatic soft-tissue sarcoma (PALETTE): a randomised, double-blind, placebo-controlled phase 3 trial. Lancet. 2012;379:1879–86. 10.1016/S0140-6736(12)60651-5 22595799 10.1016/S0140-6736(12)60651-5

[11] Schoffski P, Chawla S, Maki RG, Italiano A, Gelderblom H, Choy E, et al. Eribulin versus dacarbazine in previously treated patients with advanced liposarcoma or leiomyosarcoma: a randomised, open-label, multicentre, phase 3 trial. Lancet. 2016;387:1629–37. 10.1016/S0140-6736(15)01283-0 26874885 10.1016/S0140-6736(15)01283-0

[12] Zhou M, Bui N, Bolleddu S, Lohman M, Becker HC, Ganjoo K. Nivolumab plus ipilimumab for soft tissue sarcoma: a single institution retrospective review. Immunotherapy. 2020;12:1303–12. 10.2217/imt-2020-0155 32967520 10.2217/imt-2020-0155

[13] Wilky BA, Trucco MM, Subhawong TK, Fluoro V, Park W, Kwon D, et al. Axitinib plus pembrolizumab in patients with advanced sarcomas including alveolar soft-part sarcoma: a single-centre, single-arm, phase 2 trial. Lancet Oncol. 2019;20:837–48. 10.1016/S1470-2045(19)30153-6 31078463 10.1016/S1470-2045(19)30153-6

[14] Tawbi HA, Burgess M, Bolejack V, van Tine BA, Schetze M, Hu J, et al. Pembrolizumab in advanced soft-tissue sarcoma and bone sarcoma (SARC028): a multicentre, two-cohort, single-arm, open-label, phase 2 trial. Lancet Oncol. 2017;18:1493–501. 10.1016/S1470-2045(17)30624-1 28988646 10.1016/S1470-2045(17)30624-1 PMC7939029

[15] Saerens M, Brusselaers N, Rottey S, Decruyenaere A, Creytens D, Lapeire L. Immune checkpoint inhibitors in treatment of soft-tissue sarcoma: a systematic review and meta-analysis. Eur J Cancer. 2021;152:165–82. 10.1016/j.ejca.2021.04.034 34107450 10.1016/j.ejca.2021.04.034

[16] Italiano A, Bellera C, D’Angelo S. PD1/PD-L1 targeting in advanced soft-tissue sarcomas: a pooled analysis of phase II trials. J Hematol Oncol. 2020;13:55. 10.1186/s13045-020-00891-5 32430039 10.1186/s13045-020-00891-5 PMC7236113

[17] Florou V, Rosenberg AE, Wieder E, Komanduri KV, Kolonias D, Uduman M, et al. Angiosarcoma patients treated with immune checkpoint inhibitors: a case series of seven patients from a single institution. J Immunother Cancer. 2019;7:213. 10.1186/s40425-019-0689-7 31395100 10.1186/s40425-019-0689-7 PMC6686562

[18] Ravi V, Subramaniam A, Zheng J, Amini B, Trinh BA, Joseph J, et al. Clinical activity of checkpoint inhibitors in angiosarcoma: a retrospective cohort study. Cancer. 2022;128:3383–91. 10.1002/cncr.34370 35792683 10.1002/cncr.34370

[19] Wagner MJ, Othus M, Patel SP, Ryan C, Sangal A, Powers, B, et al. Multicenter phase II trial (SWOG S1609, cohort 51) of ipilimumab and nivolumab in metastatic or unresectable angiosarcoma: a substudy of dual anti-CTLA-4 and anti-PD-1 blockade in rare tumors (DART). J Immunother Cancer. 2021;9:e002990. 10.1136/jitc-2021-002990 10.1136/jitc-2021-002990 PMC8330584 34380663

[20] Nassif EF, Auclin E, Bahleda R, Honore C, Mir O, Dumont S, et al. TP53 mutation as a prognostic and predictive marker in sarcoma: pooled analysis of MOSCATO and ProfiLER precision medicine trials. Cancers (Basel). 2021;13:3362. 10.3390/cancers13133362 10.3390/cancers13133362 PMC8268242 34282771

[21] Ohnstad HO, Castro R, Sun J, Heintz K, Vassilev LT, Berkehagen B, et al. Correlation of TP53 and MDM2 genotypes with response to therapy in sarcoma. Cancer. 2013;119:1013–22. 10.1002/cncr.27837 23165797 10.1002/cncr.27837

[22] Bui NQ, Przybyl J, Trabucco SE, Framptom G, Hastie T, van de Rijn M, et al. A clinico-genomic analysis of soft tissue sarcoma patients reveals CDKN2A deletion as a biomarker for poor prognosis. Clin Sarcoma Res. 2019;9:12. 10.1186/s13569-019-0122-5 31528332 10.1186/s13569-019-0122-5 PMC6739971

[23] Voss RK, Callegaro D, Chiang YJ, Fiore M, Miceli R, Keung EZ, et al. Sarculator is a good model to predict survival in resected extremity and trunk sarcomas in US patients. Ann Surg Oncol. 2022. 10.1245/s10434-022-11442-2 10.1245/s10434-022-11442-2 35224688

[24] Pasquali S, Palmerini E, Quagliuolo V, Martin-Broto J, Lopez-Pousa A, Grignani G, et al. Neoadjuvant chemotherapy in high-risk soft tissue sarcomas: a Sarculator-based risk stratification analysis of the ISG-STS 1001 randomized trial. Cancer. 2022;128:85–93. 10.1002/cncr.33895 34643947 10.1002/cncr.33895

[25] Pan M, Zhou MY, Jiang C, Zhang Z, Bui N, Bien J, et al. Sex-dependent prognosis of patients with advanced soft tissue sarcoma. Clin Cancer Res. 2023. 10.1158/1078-0432.CCR-23-1990 10.1158/1078-0432.CCR-23-1990 PMC10792361 37831066

[26] Tomlins SA, Hovelson DH, Harms P, Drewery S, Falkner J, Fischer A, et al. Development and validation of StrataNGS, a multiplex PCR, semiconductor sequencing-based comprehensive genomic profiling test. J Mol Diagn. 2021;23:1515–33. 10.1016/j.jmoldx.2021.08.005 34454112 10.1016/j.jmoldx.2021.08.005

[27] Takeda M, Takahama T, Sakai K, Shimizu S, Watanabe S, Kawakami H, et al. Clinical application of the FoundationOne CDx assay to therapeutic decision-making for patients with advanced solid tumors. Oncologist. 2021;26:e588–96. 10.1002/onco.13639 33325566 10.1002/onco.13639 PMC8018334

[28] Zeng J, Johnson A, Shufean MA, Khale M, Yang D, Woodman S, et al. Operationalization of next-generation sequencing and decision support for precision oncology. JCO Clin Cancer Inform. 2019;3:1–12. 10.1200/CCI.19.00089 31550176 10.1200/CCI.19.00089 PMC6874004

[29] Beaubier N, Tell R, Lau D, Parsons J, Bush S, Perera J, et al. Clinical validation of the tempus xT next-generation targeted oncology sequencing assay. Oncotarget. 2019;10:2384–96. 10.18632/oncotarget.26797 31040929 10.18632/oncotarget.26797 PMC6481324

[30] White T, Szelinger S, LoBello J, King A, Aldrich J, Garinger N, et al. Analytic validation and clinical utilization of the comprehensive genomic profiling test, GEM ExTra((R)). Oncotarget. 2021;12:726–39. 10.18632/oncotarget.27945 33889297 10.18632/oncotarget.27945 PMC8057276

[31] Pan M, Jiang C, Tse P, Achacoso N, Alexeeff S, Solorzano AV, et al. TP53 gain-of-function and non-gain-of-function mutations are differentially associated with sidedness-dependent prognosis in metastatic colorectal cancer. J Clin Oncol. 2022;40:171–9. 10.1200/JCO.21.02014 34843402 10.1200/JCO.21.02014 PMC8718185

[32] Hernan MA, Sauer BC, Hernandez-Diaz S, Platt R, Shrier I. Specifying a target trial prevents immortal time bias and other self-inflicted injuries in observational analyses. J Clin Epidemiol. 2016;79:70–5. 10.1016/j.jclinepi.2016.04.014 27237061 10.1016/j.jclinepi.2016.04.014 PMC5124536

[33] Vidotto T, Melo CM, Castelli E, Koti M, Dos Reis RB, Squire JA. Emerging role of PTEN loss in evasion of the immune response to tumours. Br J Cancer. 2020;122:1732–43. 10.1038/s41416-020-0834-6 32327707 10.1038/s41416-020-0834-6 PMC7283470

[34] Vidotto T, Melo CM, Lautert-Dutra W, Chaves LP, Reis RB, Squire JA. Pan-cancer genomic analysis shows hemizygous PTEN loss tumors are associated with immune evasion and poor outcome. Sci Rep. 2023;13:5049. 10.1038/s41598-023-31759-6 36977733 10.1038/s41598-023-31759-6 PMC10050165

[35] Ricci R, Maggiano N, Castri F, Rinelli A, Murazio M, Pacelli F, et al. Role of PTEN in gastrointestinal stromal tumor progression. Arch Pathol Lab Med. 2004;128:421–5. 10.5858/2004-128-421-ROPIGS 15043466 10.5858/2004-128-421-ROPIGS

[36] Pan M, Jiang C, Zhang Z, Achacoso N, Alexeeff S, Solorzano AV, et al. TP53 gain-of-function and non-gain-of-function mutations are associated with differential prognosis in advanced pancreatic ductal adenocarcinoma. JCO Precis Oncol. 2023;7:e2200570. 10.1200/PO.22.00570 37163715 10.1200/PO.22.00570

[37] Lamm W, Natter C, Schur S, Kostler WJ, Reinthaller A, Krainer M, et al. Distinctive outcome in patients with non-uterine and uterine leiomyosarcoma. BMC Cancer. 2014;14:981. 10.1186/1471-2407-14-981 25523155 10.1186/1471-2407-14-981 PMC4320583

[38] Koehler K, Liebner D, Chen JL. TP53 mutational status is predictive of pazopanib response in advanced sarcomas. Ann Oncol. 2016;27:539–43. 10.1093/annonc/mdv598 26646755 10.1093/annonc/mdv598 PMC5006122

[39] Pan M, Ganjoo K, Karam A. Rapid response of a BRCA2/TP53/PTEN-deleted metastatic uterine leiomyosarcoma to olaparib: a case report. Perm J. 2021;25:20.251. 10.7812/TPP/20.251 10.7812/TPP/20.251 PMC8784049 33970096

[40] Turner NC, Huang X, Cristofanilli M. Palbociclib and fulvestrant in breast cancer. Reply. N Engl J Med. 2019;380:797. 10.1056/NEJMc1816595 30786200 10.1056/NEJMc1816595

[41] Turner NC, Slamon DJ, Ro J, Bondarenko I, Im S, Masuda N, et al. Overall survival with palbociclib and fulvestrant in advanced breast cancer. N Engl J Med. 2018;379:1926–36. 10.1056/NEJMoa1810527 30345905 10.1056/NEJMoa1810527

[42] Sledge GW Jr., Frenzel M. Analysis of overall survival benefit of abemaciclib plus fulvestrant in hormone receptor-positive, ERBB2-negative breast cancer–reply. JAMA Oncol. 2020;6:1122–3. 10.1001/jamaoncol.2020.1518 32463426 10.1001/jamaoncol.2020.1518

[43] Sledge GW Jr., Toi M, Neven P, Sohn J, Inoue K, Pivot X, et al. MONARCH 2: abemaciclib in combination with fulvestrant in women with HR+/HER2- advanced breast cancer who had progressed while receiving endocrine therapy. J Clin Oncol. 2017;35:2875–84. 10.1200/JCO.2017.73.7585 28580882 10.1200/JCO.2017.73.7585

[44] Chen S, Wu JL, Liang Y, Tang Y, Song H, Wu L, et al. Arsenic trioxide rescues structural p53 mutations through a cryptic allosteric site. Cancer Cell. 2021;39:225–39.e8. 10.1016/j.ccell.2020.11.013 33357454 10.1016/j.ccell.2020.11.013

[45] Gummlich L. ATO stabilizes structural p53 mutants. Nat Rev Cancer. 2021;21:141. 10.1038/s41568-021-00337-1 33542522 10.1038/s41568-021-00337-1

[46] Malarikova D, Berkova A, Obr A, Blahovkova B, Swaton M, Forstrova K, et al. Concurrent TP53 and CDKN2A gene aberrations in newly diagnosed mantle cell lymphoma correlate with chemoresistance and call for innovative upfront therapy. Cancers (Basel). 2020;12:2120. 10.3390/cancers12082120 10.3390/cancers12082120 PMC7466084 32751805

[47] Reis GF, Pekmezci M, Hansen HM, Rice T, Marshall R, Molinano AM, et al. CDKN2A loss is associated with shortened overall survival in lower-grade (World Health Organization Grades II-III) astrocytomas. J Neuropathol Exp Neurol. 2015;74:442–52. 10.1097/NEN.0000000000000188 25853694 10.1097/NEN.0000000000000188 PMC4397174

[48] Zheng H, Ying H, Yan H, Kimmelman AC, Hillman DJ, Chen A, et al. p53 and Pten control neural and glioma stem/progenitor cell renewal and differentiation. Nature. 2008;455:1129–33. 10.1038/nature07443 18948956 10.1038/nature07443 PMC4051433

[49] Zheng H, Ying H, Yan H, Kimmelman AC, Hillman DJ, Chen A, et al. Pten and p53 converge on c-Myc to control differentiation, self-renewal, and transformation of normal and neoplastic stem cells in glioblastoma. Cold Spring Harb Symp Quant Biol. 2008;73:427–37. 10.1101/sqb.2008.73.047 19150964 10.1101/sqb.2008.73.047

[50] Naqash AR, Floudas CS, Maoz A, Xu J, Baca Y, Zeng J, et al. STK11/TP53 co-mutated non-small cell lung cancer (NSCLC) to display a unique tumor microenvironment (TME) and metabolic profile. J Clin Oncol. 2021;39:9087. 10.1200/JCO.2021.39.15_suppl.9087

[51] Bange E, Marmarelis ME, Hwang WT, Yang, Y, Thompson JC, Rosenbarm J, et al. Impact of KRAS and TP53 co-mutations on outcomes after first-line systemic therapy among patients with STK11-mutated advanced non-small-cell lung cancer. JCO Precis Oncol. 2019;3:PO.18.00326. 10.1200/PO.18.00326 10.1200/PO.18.00326 PMC6699781 31428721

[52] Zeng J, Hills SA, Ozono E, Diffley JFX. Cyclin E-induced replicative stress drives p53-dependent whole-genome duplication. Cell. 2023;186:528–42.e14. 10.1016/j.cell.2022.12.036 36681079 10.1016/j.cell.2022.12.036 PMC7619399

