# Supplementary material for: PTEN pathogenic variants are associated with poor prognosis in patients with advanced soft tissue sarcoma
Source: BJC Rep. 2024 Jan 30;2:9. doi: 10.1038/s44276-023-00029-3 (PMC11524139; doi:10.1038/s44276-023-00029-3)

**Data supplement**

PTEN Pathogenic Variant Is Associated with Poor Prognosis in Patients with Advanced Soft Tissue Sarcoma

**Minggui Pan, MD, PhD1,2*; Maggie Y. Zhou, MD2*, Chen Jiang, PhD1; Zheyang Zhang, MS3, Nam Bui, MD2, Jeffrey Bien, MD2, Amanda Siy2, Ninah Achacoso, MS1; Aleyda V. Solorzano, MD1; Pam Tse, BA1; Elaine Chung, MPH1; Wenwei Hu, PhD^4^; Sachdev Thomas, MD^5^; and Kristen Ganjoo, MD^2;^ Laurel A. Habel, PhD^1^**

1Division of Research, Kaiser Permanente, Oakland, CA 94612

2Sarcoma Program, Division of Oncology, Stanford University School of Medicine, Stanford, CA 94305.

3State Key Laboratory of Cellular Stress Biology, School of Life Sciences, Faculty of Medicine and Life Sciences, Xiamen University; and National Institute for Data Science in Health and Medicine, Xiamen University, Xiamen, Fujian 361102, China

4Rutger’s Cancer Institute of New Jersey, New Brunswick, NJ 08903

5Department of Oncology and Hematology, Kaiser Permanente, Vallejo, CA 94589

*Co-first author

**Corresponding Author:** Minggui Pan, MD, PhD, Sarcoma Program, Division of Oncology, Stanford University School of Medicine, Stanford, CA 94304.

Email: [minggui@stanford.edu](mailto:minggui@stanford.edu)

**Table A1. Distribution of other histology subtypes.**

**Table A2. Demographics of patients with wild-type (wt) and mutant (mut) *TP53*.**

**Table A3. Demographics of patients with *TP53* hotspot and non-hotspot mutations.**

**Table A4. Demographics of patients with wild-type (wt) and mutant (mut) *CDKN2A*. Table A5. Demographics of patients with wild-type (wt) and mutant (mut) *Rb1*.**

**Table A6. Demographics of patients with wild-type (wt) and mutant (mut) *ATRX*.**

**Figure A1. Kaplan-Meyer curves of OS of four different histology subtypes.**

**Table A1. Distribution of Other histology subtypes**

| Histology | Patients (n = 214) |
| --- | --- |
| Alveolar soft part  sarcoma | 4 |
| Angiosarcoma | 46 |
| Carcinosarcoma | 12 |
| Clear cell sarcoma | 6 |
| Desmoplastic small  round cell tumor | 3 |
| Epithelioid  hemangioepithelioma | 7 |
| Epithelioid sarcoma | 5 |
| Exoskeletal  osteosarcoma | 2 |
| High-grade endometrial stromal  sarcoma | 2 |
| Intimal sarcoma | 7 |
| Malignant peripheral  nerve sheath tumor | 16 |
| Myxofibrosarcoma | 26 |
| PeComa | 4 |
| Rhabdomyosarcoma | 23 |
| Solitary fibrous tumor | 21 |
| Synovial sarcoma | 26 |
| Uterine  adenosarcoma | 4 |

**Table 2. Demographics of patients with wild-type (wt) and mutant (mut) *p53*.**

|  | | Wt (n = 306) | Mut (n = 296) | P value |
| --- | --- | --- | --- | --- |
| Median age | | 57 (19-92) | 61 (22-87) | 0.001 |
| Female | | 161 (52.6) | 189 (63.9) | 0.005 |
| Race | Asian | 51 (16.7) | 64 (21.6) | 0.008 |
|  | Black | 10 (3.3) | 15 (5.1) |  |
|  | Hispanic | 59 (19.3) | 34 (11.5) |  |
|  | White | 170 (55.6) | 177 (59.8) |  |
|  | Others | 16 (5.2) | 6 (2.0) |  |
| PS | 0-1 | 261 (85.3) | 261 (88.2) | 0.44 |
|  | 2-4 | 33 (10.8) | 23 (7.8) |  |
|  | Unknown | 12 (3.9) | 13 (4.0) |  |
| CCI | | 1 (0-9) | 1 (0-9) | 0.77 |
| Treatment | Yes | 241 (78.8) | 233 (78.7) | 0.93 |
|  | No | 65 (21.2) | 63 (21.3) |  |
| Histology | LMS | 41 (23.6) | 133 (76.4) | < .001 |
|  | UPS | 55 (40.4) | 81 (59.6) |  |
|  | LPS | 66 (84.6) | 12 (15.4) |  |
|  | Others | 144 (67.3) | 70 (32.7) |  |
| *CDKN2A* | wt | 199 (65.0) | 264 (89.2) | < .001 |
|  | mut | 107 (35.0) | 32 (10.8) |  |
| *RB1* | wt | 283 (92.5) | 188 (63.5) | < .001 |
|  | mut | 23 (7.5) | 108 (36.5) |  |
| *PTEN* | wt | 294 (96.1) | 251 (84.8) | < .001 |
|  | mut | 12 (3.9) | 45 (15.2) |  |
| *ATRX* | wt | 211 (69.0) | 169 (57.1) | < .001 |
|  | mut | 14 (4.6) | 53 (17.9) |  |
|  | unknown | 81 (26.5) | 74 (25.0) |  |

Note: PS, performance status; CCI, Charlson comorbidity index; LMS, leiomyosarcoma; UPS, undifferentiated pleomorphic sarcoma; LPS, liposarcoma. The number inside the parenthesis represents percent except for median age

**Table A3. Demographics of patients with *p53* hotspot and non-hotspot mutations.**

|  | | | Hotspot (n = 22) | Non-hotspot (n = 274) | *P* value |
| --- | --- | --- | --- | --- | --- |
| Median age | | | 63 (34-87) | 65 (23-97) | .19 |
| Female | | | 14 (63.6) | 175 (63.9) | .98 |
| Race | | Asian | 2 (9.1) | 62 (22.6) | .24 |
|  |  | Black | 0 | 15 (5.5) |  |
|  |  | Hispanic | 2 (9.1) | 32 (11.7) |  |
|  |  | White | 18 (81.8) | 159 (58.0) |  |
|  |  | Others | 0 | 6 (2.2) |  |
| PS | 0-1 | | 21 (95.5) | 240 (87.6) | .37 |
|  | 2-4 | | 0 | 23 (8.4) |  |
|  | Unknown | | 1 (4.5) | 11 (4.0) |  |
| CCI | | | 1.5 (0-5) | 1 (0-9) | .64 |
| Treatment | | Yes | 14 (63.6) | 219 (79.9) | .07 |
|  |  | No | 8 (36.4) | 55 (20.1) |  |
| Histology | | LMS | 8 (36.4) | 125 (45.6) | .25 |
|  |  | UPS | 4 (18.2) | 77 (28.1) |  |
|  |  | LPS | 1 (4.5) | 11 (4.0) |  |
|  |  | Others | 9 (40.9) | 61 (22.3) |  |
| *CDKN2A* | wt | | 17 (67.3) | 247 (90.1) | .06 |
|  | mut | | 5 (22.7) | 27 (9.9) |  |
| *RB1* | wt | | 15 (68.2) | 173 (63.1) | .64 |
|  | mut | | 7 (31.8) | 101 (36.9) |  |
| *PTEN* | wt | | 20 (90.9) | 231 (84.7) | .41 |
|  | mut | | 2 (9.1) | 43 (15.7) |  |
| *ATRX* | wt | | 11 (50.0) | 158 (57.7) | .43 |
|  | mut | | 3 (13.6) | 50 (18.2) |  |
|  | unknown | | 8 (36.4) | 66 (24.1) |  |

Note: PS, performance status; CCI, Charlson comorbidity index; LMS, leiomyosarcoma;

UPS, undifferentiated pleomorphic sarcoma; LPS, liposarcoma.

**Table A4. Demographics of patients with wild-type (wt) and mutant (mut) *CDKN2A*.**

|  | | Wt (n = 463) | Mut (n = 139) | *P* |
| --- | --- | --- | --- | --- |
| Median age | | 57 (19-92) | 63 (22-87) | < .001 |
| Female | | 284 (61.3) | 66 (47.5) | 0.003 |
| Race | Asian | 87 (18.8) | 28 (20.1) | 0.70 |
|  | Black | 19 (4.1) | 6 (4.3) |  |
|  | Hispanic | 74 (16.0) | 19 (13.7) |  |
|  | White | 262 (56.6) | 85 (65.1) |  |
|  | Others | 21 (4.5) | 1 (.7) |  |
| PS | 0-1 | 407 (87.9) | 115 (82.7) | 0.28 |
|  | 2-4 | 39 (8.4) | 17 (12.2) |  |
|  | Unknown | 17 (3.7) | 7 (5.0) |  |
| CCI | | 1 (0-9) | 2 (0-9) | < .001 |
| Treatment | Yes | 385 (83.2) | 89 (64.0) | < .001 |
|  | No | 78 (16.8) | 50 (36.0) |  |
| Histology | LMS | 166 (35.9) | 8 (5.8) | < .001 |
|  | UPS | 100 (21.6) | 36 (25.9) |  |
|  | LPS | 20 (4.3) | 58 (41.7) |  |
|  | Others | 177 (38.2) | 37 (26.6) |  |
| *TP53* | wt | 199 (43.0) | 107 (77.0) | < .001 |
|  | mut | 264 (57.0) | 32 (23.0) |  |
| *RB1* | wt | 334 (72.1) | 137 (98.6) | < .001 |
|  | mut | 129 (27.9) | 2 (1.4) |  |
| *PTEN* | wt | 410 (88.6) | 135 (97.1) | 0.002 |
|  | mut | 53 (11.4) | 4 (2.9) |  |
| *ATRX* | wt | 281 (60.7) | 99 (71.2) | < .02 |
|  | mut | 60 (13.0) | 7 (5.0) |  |
|  | unknown | 122 (26.3) | 33 (23.7) |  |

Note: PS, performance status; CCI, Charlson comorbidity index; LMS, leiomyosarcoma; UPS, undifferentiated pleomorphic sarcoma; LPS, liposarcoma. The number inside the parenthesis represents percent except for median age.

**Table A5. Demographics of patients with wild-type (wt) and mutant (mut) *Rb1*.**

|  | | Wt (n = 471) | Mut (n = 131) | *P* |
| --- | --- | --- | --- | --- |
| Median age | | 59 (19-90) | 57 (31-92) | 0.92 |
| Female | | 263 (55.8) | 87 (66.4) | 0.03 |
| Race | Asian | 92 (19.5) | 23 (17.6) | 0.70 |
|  | Black | 20 (4.2) | 5 (3.8) |  |
|  | Hispanic | 75 (19.3) | 18 (11.8) |  |
|  | White | 265 (56.3) | 82 (62.6) |  |
|  | Others | 19 (4.0) | 3 (2.3) |  |
| PS | 0-1 | 408 (86.4) | 114 (87.0) | 0.86 |
|  | 2-4 | 45 (9.6) | 11 (8.4) |  |
|  | Unknown | 18 (3.8) | 6 (4.6) |  |
| CCI | | 1 (0-9) | 1 (0-7) | 0.01 |
| Treatment | Yes | 360 (76.4) | 114 (87.0) | 0.007 |
|  | No | 111 (23.6) | 17 (13.0) |  |
| Histology | LMS | 96 (20.4) | 78 (59.5) | < .001 |
|  | UPS | 109 (23.1) | 27 (20.6) |  |
|  | LPS | 69 (14.6) | 9 (6.9) |  |
|  | Others | 197 (41.8) | 17 (13.0) |  |
| *CDKN2A* | wt | 334 (70.9) | 129 (98.5) | < .001 |
|  | mut | 137 (29.1) | 2 (1.5) |  |
| *TP53* | wt | 283 (60.1) | 23 (17.6) | < .001 |
|  | mut | 188 (39.1) | 108 (82.4) |  |
| *PTEN* | wt | 440 (93.4) | 105 (80.2) | < .001 |
|  | mut | 31 (6.6) | 26 (19.8) |  |
| *ATRX* | wt | 317 (67.3) | 63 (48.1) | < .001 |
|  | mut | 30 (6.4) | 37 (28.2) |  |
|  | unknown | 124 (26.3) | 31 (23.7) |  |

Note: PS, performance status; CCI, Charlson comorbidity index; LMS, leiomyosarcoma; UPS, undifferentiated pleomorphic sarcoma; LPS, liposarcoma. The number inside the parenthesis represents percent except for median age.

**Table A6. Demographics of patients with wild-type (wt) and mutant (mut) *ATRX*.**

|  | | Wt (n = 380) | Mut (n = 67) | Unknown (n = 155) | *P* |
| --- | --- | --- | --- | --- | --- |
| Median age | | 58 (19-92) | 49 (37-68) | 60 (20 – 90) | 0.17 |
| Female | | 212 (55.8) | 47 (70.1) | 91 (58.7) | 0.09 |
| Race | Asian | 80 (21.1) | 17 (25.4) | 18 (11.6) | < 0.001 |
|  | Black | 14 (3.7) | 1 (1.5) | 10 (6.5) |  |
|  | Hispanic | 46 (12.1) | 7 (10.4) | 40 (25.8) |  |
|  | White | 221 (58.2) | 42 (62.7) | 84 (54.2) |  |
|  | Others | 19 (5.0) | 0 (0) | 3 (1.9) |  |
| PS | 0-1 | 329 (86.6) | 63 (94.0) | 130 (83.9) | 0.13 |
|  | 2-4 | 39 (10.3) | 2 (3.0) | 15 (9.7) |  |
|  | Unknown | 12 (3.2) | 2 (3.0) | 10 (6.5) |  |
| CCI | | 1 (0-9) | 1 (0-7) | 0 (0-6) | < 0.001 |
| Treatment | Yes | 296 (77.9) | 56 (83.6) | 122 (78.7) | 0.58 |
|  | No | 84 (22.1) | 11 (16.4) | 33 (21.3) |  |
| Histology | LMS | 85 (22.4) | 34 (50.7) | 55 (35.5) | < 0.001 |
|  | UPS | 96 (25.3) | 14 (20.9) | 26 (16.8) |  |
|  | LPS | 52 (13.7) | 5 (7.5) | 21 (13.5) |  |
|  | Others | 147 (38.7) | 14 (20.9) | 53 (34.2) |  |
| *RB1* | wt | 317 (83.4) | 30 (44.8) | 124 (80.0) | < .001 |
|  | mut | 63 (16.6) | 37 (55.2) | 31 (20.0) |  |
| *TP53* | wt | 211 (55.5) | 14 (20.9) | 81 (52.3) | < 0.001 |
|  | mut | 169 (44.5) | 53 (79.1) | 74 (47.7) |  |
| *CDKN2A* | wt | 281 (73.9) | 60 (89.6) | 122 (78.7) | 0.017 |
|  | mut | 99 (26.1) | 7 (10.4) | 33 (21.3) |  |
| *PTEN* | wt | 349 (91.8) | 58 (86.6) | 138 (89.0) | 0.30 |
|  | mut | 31 (8.2) | 9 (13.4) | 17 (11.0) |  |

Note: PS, performance status; CCI, Charlson comorbidity index; LMS, leiomyosarcoma; UPS, undifferentiated pleomorphic sarcoma; LPS, liposarcoma. The number inside the parenthesis represents percent except for median age.

**Figure A1. Forest lot of adjusted hazard ratios of OS among patients with other histologic subtypes (Other).** aHR, adjusted hazard ratio; OS, overall survival; Mut, mutation; Wt, wild-type.


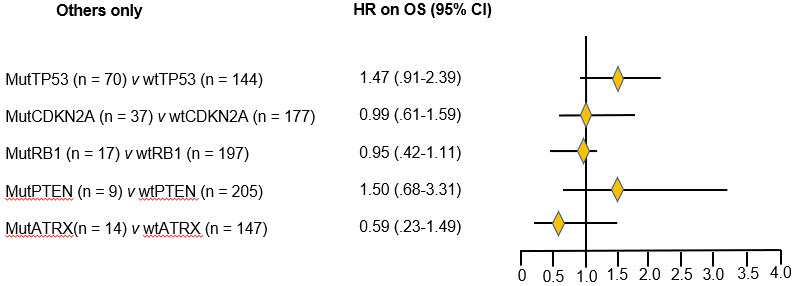

Supplement: Supplementary file 1 — Data supplement [file 44276_2023_29_MOESM1_ESM.docx]
